# A Case of Laparoscopic-Assisted Percutaneous Endoscopic Gastrostomy (LAPEG) for Gastric Volvulus

**DOI:** 10.1155/2019/3468084

**Published:** 2019-12-03

**Authors:** Shuhei Ueda, Hajime Orita, Tomoaki Ito, Satoshi Tokuda, Shunsuke Sakuraba, Tomoyuki Kushida, Mutsumi Sakurada, Hiroshi Maekawa, Koichi Sato

**Affiliations:** Department of Surgery, Juntendo Shizuoka Hospital, Juntendo University School of Medicine, Shizuoka, Japan

## Abstract

**Background:**

Percutaneous endoscopic gastrostomy (PEG) is the standard modality for long-term enteral nutrition for patients with dysphagia. Compared with open gastrostomy, though PEG is an extremely safe procedure with fewer complications, there are severe cases due to anatomical features. For these cases, laparoscopic-assisted percutaneous endoscopic gastrostomy (LAPEG) is the optimal method.

**Case Presentation:**

A 52-year-old man had a disturbance in swallowing because of cerebral infarction. We attempted PEG under gastrointestinal fiberscope (GIF) and colon ﬁberscope inspection; however, the procedure was unsuccessful because it was impossible to move the transverse colon downward. We therefore attempted LAPEG to observe the stomach and other organs. Under laparoscopic observation, we diagnosed gastric volvulus, classified as the organo-axial type. For this reason, inserting the tube through the skin was very difficult. We easily corrected the gastric volvulus by using laparoscopic forceps and were finally able to place the PEG tube safely.

**Discussion:**

Gastric volvulus is rare in clinical practice. The treatment of gastric volvulus depends on whether mucosal ischemia is present. Endoscopic reduction of gastric volvulus is effective for many patients. Surgical treatment should be considered for patients with gastric volvulus that frequently recurs. In our patient, completely inserting the GIF was impossible; therefore, we could not correctly diagnose gastric volvulus. Laparoscopy-assisted PEG is a useful and safe technique for placing a gastrostomy tube in patients presenting with anatomical difficulties. Moreover, in our patient, gastropexy was performed with PEG. Therefore, LAPEG may be used to prevent the recurrence of gastric volvulus. Gastropexy is a useful option in LAPEG.

**Conclusions:**

Laparoscopy has the advantage of allowing a direct inspection of the stomach while gastrostomy is performed and may reveal complications to PEG insertion. Furthermore, in our patient, gastropexy was performed with PEG.

## 1. Introduction

Percutaneous endoscopic gastrostomy (PEG) was introduced in the 1980s [1, 2]. It is the standard modality for long-term enteral nutrition for patients with dysphagia [3]. Percutaneous endoscopic gastrostomy is a safe procedure and has fewer complications than does open gastrostomy [4]. Most complications are minor. However, less common major complications that laparotomy requested may be fatal such as perforation of the colon and severe bleeding [5, 6]. For susceptible patients, surgical intervention may be required to avoid misplacing the tube into the colon [5]. The reasons for challenging situations involve complex medical issues such as postsurgical anatomy and obesity. These issues are commonly secondary to the inability to transilluminate the abdominal wall. Complex medical issues include the transverse colon flopping over the stomach and gastric volvulus [7, 8].

Gastric volvulus is the rotation of the stomach along its short or long axis, which causes variable degrees of gastric inlet and outlet obstruction [9]. Volvulus is more common in children; however, a few reports of volvulus in adults exist [10].

To encourage the use of minimum invasive surgery, some reports of laparoscopic-assisted PEG (LAPEG) have described its advantages for difficult cases of PEG insertion, instead of laparotomy association [11–13].

In this paper, in cases who have not shown positive transillumination through the abdominal wall, we report a case of gastric volvulus undergone LAPEG because of the failure of normal PEG procedure. In addition, we demonstrated the advantage of LAPEG for patients with gastric volvulus for whom the usual PEG procedure is difficult.

## 2. Case Presentation

A 52-year-old man was admitted to our hospital for PEG tube placement. The patient's body mass index was 19.0 kg/m^2^. He had previously been treated for acute aortic dissection of the aortic arch and descending aorta under thoracotomy. A cardiologist had replaced the aortic arch and descending aorta. After the operation, he experienced a disturbance in swallowing because of cerebral infarction. For 2 years, he had been experiencing occasional vomiting. Blood examination and laboratory serum tests did not reveal any abnormal findings.

**Figure 1:**
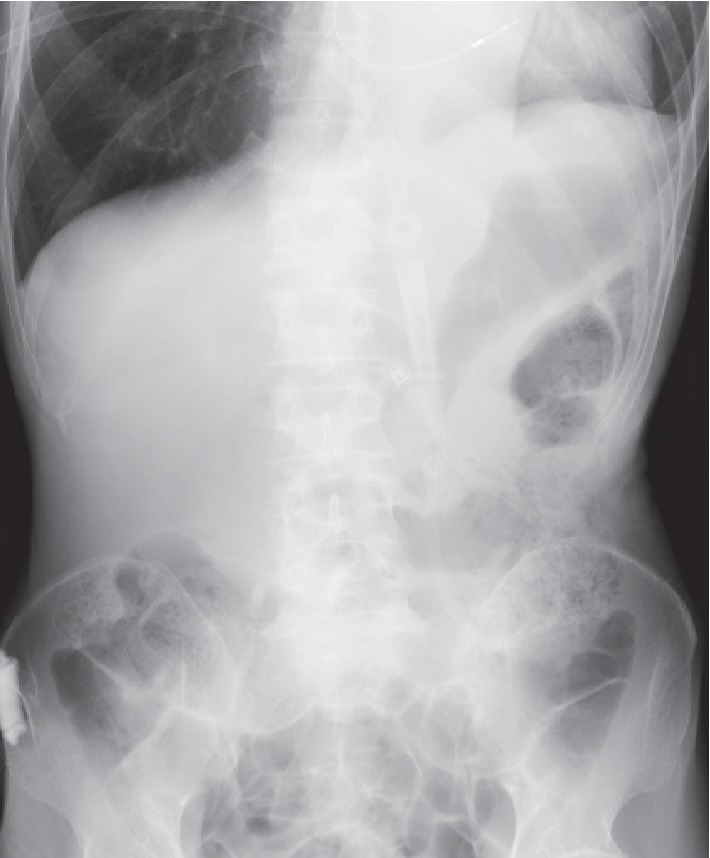
The X-ray image does not show a distended stomach.

**Figure 2:**
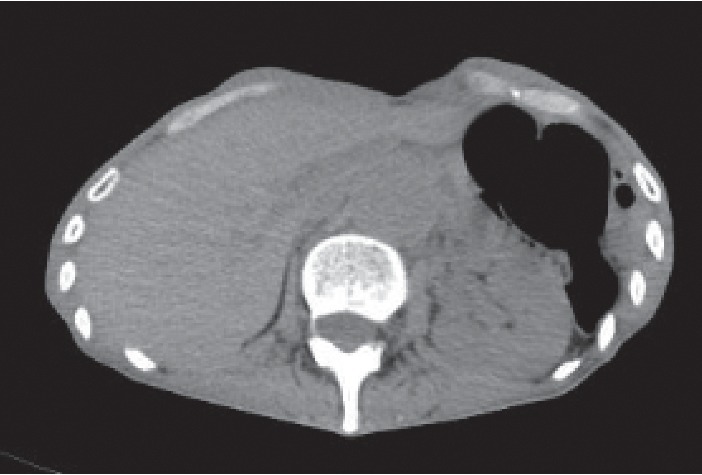
The computed tomography image does not show a distended stomach.

X-ray and computed tomography (CT) scan images did not show a distended stomach (Figures 1 and 2). The CT examination revealed that the transverse colon was on the entire surface of the stomach, and therefore PEG insertion would be difficult.

We first attempted PEG under gastrointestinal fiberscope (GIF) inspection. The GIF findings indicated no remarkable abnormalities. The gastric folds were smooth and not tortuous. We failed to place a PEG tube while using a GIF because the abdominal wall could not be transilluminated. Therefore, we believed that the transverse colon was covering the stomach.

**Figure 3:**
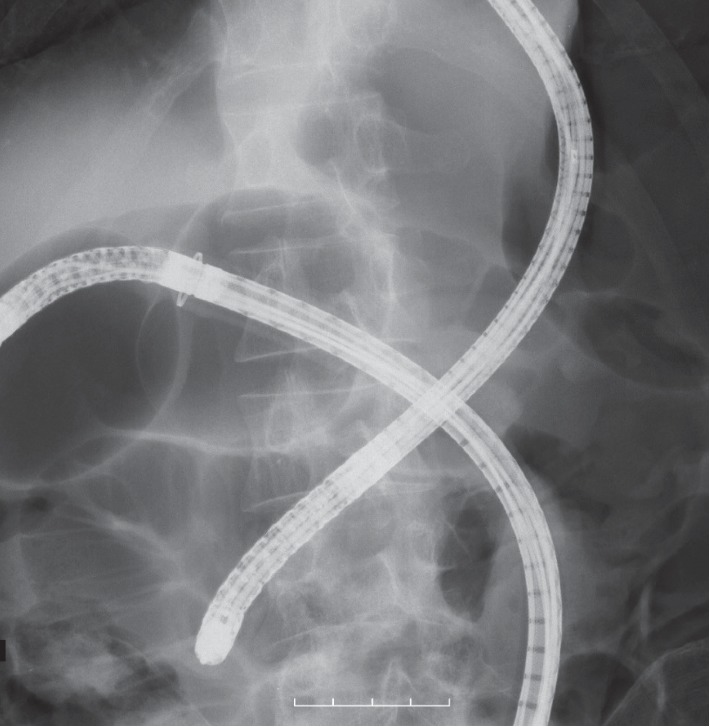
The X-ray image of the collaborative work with the gastrointestinal fiberscope and colon fiberscope. We abandoned the attempt to insert the PEG because the transverse colon is flopped over the stomach.

We then attempted PEG insertion by using a colon fiberscope to move the transverse colon down to the caudal side; however, this procedure was unsuccessful (Figure 3). The reasons for the unsuccessful PEG tube insertion were that we failed to move the transverse colon down and we could not detect transillumination of the abdominal wall. We subsequently attempted LAPEG to observe the stomach and other organs because we believed some abnormalities around the stomach might have existed.

**Figure 4:**
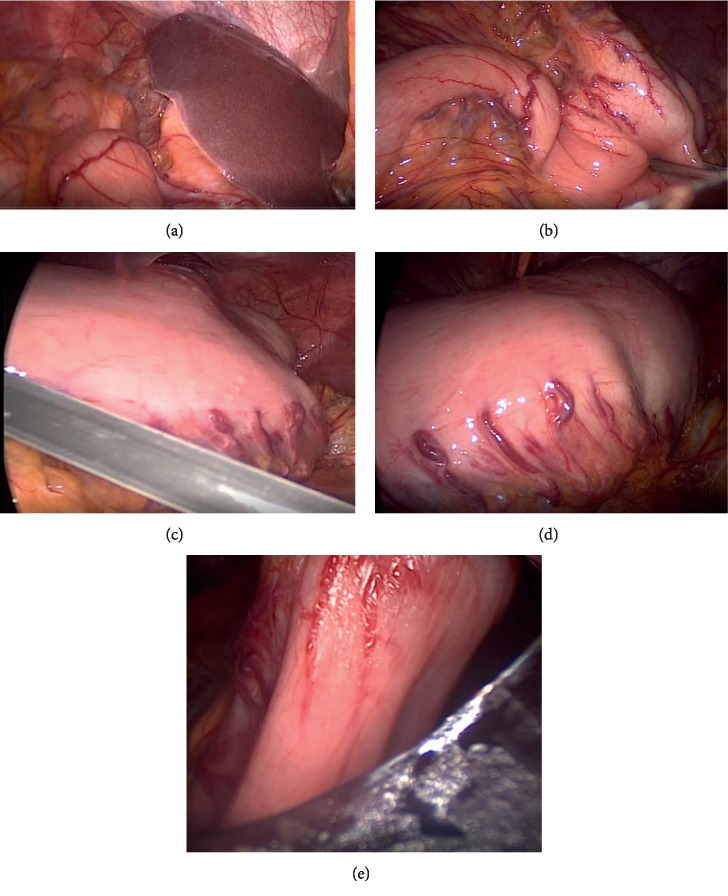
The progress status during laparoscopic surgery. (a) The diagnosis and (b, c) the untwisting of the gastric volvulus. Under laparoscopic observation, we diagnosed organo-axial type of gastric volvulus. (d) We easily corrected gastric volvulus by using laparoscopic forceps and (e) finally placed the PEG tube safely.

Laparoscopic surgery was performed under general anesthesia. Based on laparoscopic observation, we determined that the stomach was twisted and the transverse colon was not on the stomach. By using laparoscopy, we found a greater curvature of the stomach on the head side (i.e., the stomach was twisted 180 degrees). We diagnosed the patient as having the organo-axial type of gastric volvulus. During the endoscopy procedure, we were unable to derotate the stomach to its original position although the stomach had no adhesions to neighboring organs and paraesophageal hernia. We easily corrected gastric volvulus by using laparoscopic forceps and derotating the stomach. We then sutured the anterior wall of gastric body to the abdominal wall at four sites by using the Funada-style gastropexy kit [14]. We finally inserted the PEG tube safely at the anterior wall of the gastric body (Figure 4). The operative time was 62 minutes.

After the PEG tube insertion, the patient recovered smoothly and was discharged from the hospital without any complications. The patient has not had a recurrence of gastric volvulus. We were able to place the tube safely without interfering with organs surrounding the stomach by using the laparoscopy procedure.

## 3. Discussion

Gastric volvulus is rare in clinical practice. It is defined as an abnormal rotation of the stomach of more than 180 degree [4, 15]. Gastric volvulus is classified, based on the axis of torsion, as organo-axial type, mesentero-axial type, and combined type [15]. The cause of gastric volvulus is classified as primary or secondary, and the clinical course of patients with gastric volvulus is acute or chronic recurrent [15].

Acute gastric volvulus presents as a surgical emergency with epigastric pain, inability to vomit, and epigastric distention; by contrast, chronic gastric volvulus is often associated with intermittent postprandial epigastric distention [1, 16]. Chronic gastric volvulus is often diagnosed correctly because the patients do not have remarkable symptoms.

A CT scan or endoscopic examination is useful to diagnose abnormal position and torsion of the stomach [1, 4, 15]. The treatment of gastric volvulus depends on whether mucosal ischemia is present. Endoscopic reduction of gastric volvulus is effective in many patients [1, 4]. If gastric volvulus recurs, surgical treatment is necessary.

In our patient, we could not correctly diagnose gastric volvulus. However, laparoscopic findings revealed gastric volvulus, which explained why the PEG tube placement was unsuccessful. Various techniques exist for gastrostomy tube placement; among these, PEG is the most standard procedure. However, a large meta-analysis of PEG reported an overall complication rate of 9.2%, morbidity rate of 9.4%, and mortality rate of 0.53% [16–18]. The most fatal and risky complication is perforation of the colon or other organs [16]. Laparoscopy is used to avoid such fatal complications in difficult cases.

Laparoscopy has the advantage of allowing a surgeon to directly inspect the stomach while performing a gastrostomy [1]. Furthermore, laparoscopy may reveal hindrances to PEG insertion and LAPEG minimizes the risk of complications. Laparoscopic-assisted PEG is a useful and safe technique for placing a gastrostomy tube. It also aids in determining why a PEG insertion has failed. Moreover, in our patient, gastropexy was performed with PEG. Gastropexy may avoid twisting of the stomach. Thus, LAPEG may prevent the recurrence of gastric volvulus. Gastropexy is a useful option in LAPEG.

## 4. Conclusion

Laparoscopic-assisted PEG appears to be a safe and useful procedure for gastropexy in patients with dysphagia and gastric volvulus complications.
